# Acute Effect of Vibration Roller With and Without Rolling on Various Parts of the Plantar Flexor Muscle

**DOI:** 10.3389/fphys.2021.716668

**Published:** 2021-09-22

**Authors:** Masatoshi Nakamura, Shigeru Sato, Ryosuke Kiyono, Riku Yoshida, Yuta Murakami, Koki Yasaka, Kaoru Yahata, Andreas Konrad

**Affiliations:** ^1^Institute for Human Movement and Medical Sciences, Niigata University of Health and Welfare, Niigata, Japan; ^2^Department of Physical Therapy, Niigata University of Health and Welfare, Niigata, Japan; ^3^Institute of Human Movement Science, Sport and Health, University of Graz, Graz, Austria

**Keywords:** shear elastic modulus, maximal voluntary isometric contraction, concentric strength, drop jump, foam rolling

## Abstract

A single use of a vibration foam roller likely increases the range of motion (ROM) without decreasing muscle strength and athletic performance. However, to date, no study compared the effects of a vibration roller with and without rolling on various parts of the plantar flexor muscle. Therefore, this study aimed to compare the effects of the vibration foam roller with rolling or without rolling at the muscle-tendon junction (MTJ) or the muscle belly on dorsiflexion (DF) ROM, passive torque at DF ROM, shear elastic modulus, muscle strength, and jump performance. Fifteen healthy young males performed the following three conditions: (1) vibration rolling over the whole muscle-tendon unit, (2) static vibration on muscle belly, and (3) static vibration on MTJ for three-set 60-s vibration in random order. In this study, DF ROM, passive torque, shear elastic modulus, muscle strength, and single-leg drop jump were measured before and immediately after the interventions. The DF ROM and passive torque at DF ROM were increased after all three conditions, whereas the shear elastic modulus was decreased after vibration rolling and static vibration on the muscle belly, but not following static vibration of the MTJ. In addition, there were no significant changes in muscle strength and jump performance in any group. Our results showed that vibration with rolling or static vibration on muscle belly could be effective to improve ROM and muscle stiffness without adverse effects of muscle strength and athletic performance.

## Introduction

In sports and rehabilitation settings, various approaches are performed to increase joint flexibility [i.e., range of motion (ROM)] and decrease passive muscular stiffness. Static stretching is a typical approach; however, in recent years, self-massage treatment using foam rollers, sticks, or balls has become a popular technique to increase ROM. In addition, a meta-analysis study indicated foam rolling (FR) intervention as effective as static stretching to increase ROM (Wilke et al., 2020) without decreasing muscle strength or athletic performance (Sullivan et al., 2013; Lee et al., 2018; Wiewelhove et al., 2019; Phillips et al., 2021). As reported in many previous studies, certain duration static stretching intervention could decrease the muscle strength, the so-called stretch-induced force deficit (Kay and Blazevich, 2012; Behm et al., 2016; Konrad et al., 2021). Thus, FR intervention is considered as an approach that could increase flexibility without decreasing muscle strength.

With regard to the effect of FR intervention on muscle stiffness, it could decrease muscle stiffness in the hamstring (Morales-Artacho et al., 2017) or anterior thigh (Baumgart et al., 2019) and no changes in muscle stiffness (Baumgart et al., 2019; Nakamura et al., 2021a,b) or hardness (Yoshimura et al., 2020) in medial gastrocnemius (MG) muscle. However, Reiner et al. (2021) showed that vibration FR intervention could decrease muscle stiffness rather than FR intervention without vibration (Reiner et al., 2021). No consensus exists on the superior effect of vibration FR intervention to FR without vibration (García-Gutiérrez et al., 2018; Lee et al., 2018); however, a meta-analysis has speculated that vibration FR interventions could induce a greater increase in ROM than FR intervention alone based on less evidence (Wilke et al., 2020). A possible superior effect of vibration FR intervention over FR intervention alone could be attributed to greater changes in mechanoreceptors due to vibration (Behm and Wilke, 2019).

Generally, FR interventions are performed by using rolling foam rollers, sticks, or balls; however, achieving performance safety is difficult, especially when using foam rollers with vibration. A previous study by Wilke et al. (2018) compared rolling and static compression (without vibration) of myofascial trigger points, which showed that only static compression of myofascial trigger points increased the pain pressure threshold. Thus, it can be assumed that when using a vibration foam roller, only static compression of the vibration foam roller could effectively increase ROM and decrease muscle stiffness. In addition, previous studies investigating the massage effect showed that intervention near the muscle-tendon junction (MTJ) has greater ROM improvement rather than intervention on the muscle belly (Huang et al., 2010; Akazawa et al., 2016). Akazawa et al. (2016) suggested that massage intervention near the MTJ might affect neighboring neural pathways and decrease stress on the peripheral nerves, which may contribute to the change in stretch tolerance. Also, our previous studies showed that the increase in ROM after FR intervention could be related to a change in stretch tolerance, not to a change in muscle stiffness (Nakamura et al., 2021a,b). Therefore, static compression caused by vibration is possibly more effective for an increase in ROM (but likely not for a decrease in muscle stiffness) at MTJ than at the muscle belly.

Thus, this study aimed to compare the effects of vibration with rolling and static vibration on muscle belly or MTJ on dorsiflexion (DF) ROM, passive torque at DF ROM, the shear elastic modulus of MG, muscle strength [maximal voluntary isometric (MVC-ISO), concentric contraction torque (MVC-CON)], and jump performance.

## Methods

### Experimental Design

A randomized repeated measures experimental design was used to compare the effects of (1) vibration rolling (on the whole muscle-tendon unit) and (2) static vibration on muscle belly or vibration on MTJ on DF ROM, passive torque at ROM, the shear elastic modulus of MG, muscle strength, and single-leg drop jump performance in the dominant leg (preferred kicking ball). Participants were instructed to visit the laboratory three times with an interval of >72 h and were exposed to the following three conditions: (1) vibration rolling condition (ROLL), (2) vibration on muscle belly condition (Muscle Belly), and (3) vibration on MTJ condition (MTJ) for three-set 60-s vibration with 30-s intervals between each set. All outcome variables were measured before (PRE) and immediately after the intervention (POST). In both PRE and POST measurements, (1) DF ROM and passive torque, (2) shear elastic modulus, (3) MVC-ISO, (4) MVC-CON, and (5) single-leg drop jump performance were measured in this order.

### Participants

A total of 15 healthy untrained male adults participated in this study (age: 22.5 ± 3.1 years, height: 169.8 ± 4.8 cm, and body weight: 62.2 ± 5.3 kg). Inclusion criteria were as follows: no regular resistance training within the past 6 months, neuromuscular disease, and history of lower-limb orthopedic injuries. All participants provided written informed consent after being fully informed of the study procedures and purposes. After calculating the sample size required for a two-way repeated measures ANOVA [effect size = 0.40 (large), α error = 0.05, and power = 0.80] using G^*^ power 3.1 software (Heinrich Heine University, Düsseldorf, Germany) based on a previous study (Reiner et al., 2021), >14 participants were determined to be required in this study. This study was conducted in accordance with the Declaration of Helsinki and was approved by the Niigata University of Health and Welfare, Niigata, Japan.

### Assessment of the DF ROM and Passive Torque

Participants were secured in a seated position on the chair of an isokinetic dynamometer with a knee angle of 0° (i.e., anatomical position). Moreover, the trunk and pelvis of the participant were fixed with a belt while the participant was reclined (hip angle at 70°) to prevent tension at the back of the knee. Thereafter, the footplate of the dynamometer was passively and isokinetically dorsiflexed at a speed of 5°/s from 30° plantar flexion position to DF, stopping just before the participant started to feel discomfort or pain. Before DF ROM and passive torque assessment, two cycles of passive DF motion were performed so that participants can familiarize the procedure and prevent passive stretching to induce a conditioning effect on muscle-tendon stiffness (Konrad and Tilp, 2014; Hirata et al., 2017). After familiarization trials, participants stopped the dynamometer by activating a safety trigger when they started to feel discomfort or pain, with the angle just before this point defined as DF ROM (Nakamura et al., 2020, 2021d; Sato et al., 2020b). In addition, passive torque at DF ROM (maximum ROM angle) was defined as the stretch tolerance (Gajdosik et al., 2007; Weppler and Magnusson, 2010; Mizuno et al., 2013).

Throughout the passive DF test, participants were requested to completely relax and not make any voluntary contraction. We confirmed the absence of a voluntary contraction of the MG by monitoring the muscle activity through surface electromyography (FA-DL-720-140; 4Assist, Tokyo, Japan). Surface electrodes (Blue Sensor N, Ambu A/S, Ballerup, Denmark) were placed on the muscle belly of the MG. All data were confirmed to be collected during a relaxed state (i.e., no muscle activity exceeding 5% of the MVC-ISO) (Nakamura et al., 2011). Muscle activity was filtered using a band-pass filter at 10–1,000 Hz before being digitally stored (10-kHz sampling rate) on a personal computer for offline analysis. Analysis was performed using PowerLab 8/30 (AD Instruments, Colorado Springs, CO, United States) and LabChart 7 (AD Instruments); then, root–mean–square (RMS, 50 ms window) values were calculated.

### MG Shear Elastic Modulus Assessment

This study measured the shear elastic modulus of MG using ultrasonic shear wave elastography (Aplio 500, Toshiba Medical Systems, Tochigi, Japan) with a 5–14 MHz linear probe at a 10° DF position with a knee angle of 0°, similar to DF ROM measurement. The shear elastic modulus of MG was measured at 30% of the lower leg length from the popliteal crease to the lateral malleolus (Sato et al., 2020a; Nakamura et al., 2021b). Ultrasound image measurements were performed twice using the long-axis image of the MG. Shear wave speed analysis on ultrasound images was performed using image analysis software (MSI Analyzer version 5.0; Rehabilitation Science Research Institute, Japan). The largest possible area in MG was set as the region of interest (ROI) for shear wave speed (Vs) measurement, with the average vs. value inside the ROI being obtained. The shear elastic modulus was calculated as μ (kPa) = ρVs^2^, where ρ is muscle mass density (1,000 kg/m^3^). The average value of shear elastic modulus obtained from two ultrasound images was used for analysis.

### MVC-ISO and MVC-CON

MVC-ISO and MVC-CON torque measurements were conducted using a dynamometer, with a similar position for DF ROM and shear elastic modulus assessment. Moreover, MVC-ISO of the plantar flexors was measured at the ankle joint in the neutral position (0°). MVC-ISOs were performed for 5 s over two sets with a 60-s rest between each set. The average MVC-ISO value was used for analyses. Similarly, plantar flexor MVC-CON torque was measured from 10° of DF to 20° of plantar flexion, with an angular velocity of 30°/s. Additionally, maximal concentric contractions were performed three times in each sequence. Throughout the measurement, participants were verbally encouraged during muscle contraction to promote maximal efforts by investigators. Maximum torque was adopted over three concentric contractions as MVC-CON torque.

### Single-Leg Drop Jump Height

Single-leg drop jumps were performed from a 20-cm box onto a set of mat switches (Jump mat system; 4Assist, Tokyo, Japan). After three familiarization repetitions, three sets of single-leg drop jumps were performed and measured. For single-leg drop jump measurements, participants were instructed to step off the box and immediately perform a maximal vertical jump using the dominant side of the ankle plantar flexors only without using the knee and hip muscles upon landing. Both hands were crossed in front of the chest. The maximal vertical jump height over three jump measurements was then calculated using the flight time method.

### ROLL or Muscle Belly, or MTJ

The vibration was applied over plantar flexors using a foam roller (Vyper 2.0, Hyperice, Irvine, CA, USA) with a frequency of 48 Hz (Nakamura et al., 2021c). In all conditions, the physical therapist instructed the participants to use the foam roller and practice using the foam roller on the non-intervention leg for a familiarization session just before the intervention. Participants were instructed to perform 60-s bouts of intervention for three sets, with a 30-s rest between each set. In ROLL, one rolling cycle from the Achilles tendon to the popliteal fossa, especially on the medial portion of plantar flexors, was performed with a frequency of 15 cycles/min using a metronome (Smart Metronome; Tomohiro Ihara, Japan). In Muscle Belly and MTJ conditions, static vibration on the muscle belly of MG where is similar location for shear elastic modulus measurement and MTJ of MG, which was found by ultrasound, was performed using the same foam roll. In all three conditions, vibration foam roller intervention was performed unilaterally in a seated position with the knees extended and the ankle in a plantar flexion but relaxed. Participants were instructed to place as much body mass on the roller as tolerable.

### Test-Retest Reliability of Measurements

The test-retest reliability of the DF ROM measurement, passive torque at DF ROM, shear elastic modulus, MVIC-ISO, MVIC-CON, and drop jump height for seven healthy men (age, 22.7 ± 1.3 years; height, 170.4 ± 5.3 cm; and weight, 61.8 ± 4.4 kg) was determined using the coefficient variation (CV) and intraclass correlation coefficient (ICC), with 5-min rest interval between two measures in the similar protocol in this study. The CV of measurements for DF ROM, passive torque at DF ROM, shear elastic modulus, MVIC-ISO, MVIC-CON, and drop jump height were 3.4 ± 3.8%, 5.7 ± 3.1%, 5.8 ± 4.8%, 1.4 ± 0.6%, 2.8 ± 2.2%, and 2.9 ± 1.7%, respectively, and the ICC (1, 1) for measurements were 0.99, 0.93, 0.90, 0.98, 0.86, and 0.93, respectively.

### Statistical Analysis

Statistical analyses were conducted using Statistical Package for the Social Sciences version 24.0 (IBM Corp., Armonk, NY, USA). Skewness, kurtosis, Q-Q plots, and Shapiro-Wilk tests were assessed to confirm the normality of data distribution. For all variables, a two-way repeated measures ANOVA [time (PRE vs. POST) and condition (ROLL vs. Muscle Belly vs. MTJ)] was performed to analyze the interaction and main effects. On confirming a significant interaction or main effect, Bonferroni *post-hoc* was performed to compare PRE and POST values in each condition. Changes from PRE to POST values were calculated, and Bonferroni *post-hoc* was conducted to compare these changes if a significant interaction effect was found. The effect size was calculated as a difference in the mean value between PRE and POST divided by the pooled SD (Cohen, 1988), with an ES of 0.00–0.19, 0.20–0.49, 0.50–0.79, and ≥ 0.80 considered trivial, small, moderate, and large, respectively. A *p*-value of < 0.05 indicated statistical significance. Descriptive data were reported as mean ± SD.

## Results

Table 1 shows the changes in DF ROM, passive torque at DF ROM, and shear elastic modulus of MG at pre- and post-intervention in three conditions. The two-way repeated measures ANOVA showed no significant interaction effect for DF ROM (*F* = 1.24, *p* = 0.30, and $\eta_{\text{p}}^{2}$ = 0.081), but a main effect for time (*F* = 66.55, *p* < 0.01, and $\eta_{\text{p}}^{2}$ = 0.83). The *post-hoc* test showed a significant increase in DF ROM after all interventions (ROLL: *p* < 0.01, *d* = 0.705; muscle belly: *p* < 0.01, *d* = 0.365; and MTJ: *p* < 0.01, *d* = 0.423). Moreover, a significant interaction effect was found for passive torque at DF ROM (*F* = 6.0, *p* < 0.01, and $\eta_{\text{p}}^{2}$ = 0.284). The *post-hoc* test showed a significantly increased passive torque at DF ROM after all interventions (ROLL: *p* < 0.01, *d* = 0.956; Muscle Belly: *p* = 0.03, *d* = 0.352; and MTJ: *p* < 0.01, *d* = 0.596).

**Table 1:** Changes in dorsiflexion range of motion (DF ROM), passive torque at DF ROM, and shear elastic modulus before (PRE) and immediately after vibration rolling (ROLL) or vibration on the muscle belly (Muscle Belly) or vibration on muscle-tendon junction (MTJ) intervention (POST).

	**ROLL**		**Muscle Belly**		**MTJ**		**Interaction effect**	
	**PRE**	**POST**	**PRE**	**POST**	**PRE**	**POST**	***P*-value**	** $\eta_{\text{p}}^{2}$ **
DF ROM (°)	24.1 ± 7.5	29.4 ± 7.4^**^	25.1 ± 8.3	28.2 ± 8.4^*^	22.9 ± 8.2	26.2 ± 7.7^**^	*p* = 0.304	0.081
Effect size	d=	0.705	d=	0.365	d=	0.423		
Passive torque at DF ROM (Nm)	21.9 ± 6.3	28.4 ± 7.2^**^	24.0 ± 7.7	26.6 ± 7.4^*^	21.6 ± 7.6	26.2 ± 7.8^**^	*p* < 0.01	0.284
Effect size	d=	0.956	d=	0.352	d=	0.596		
Shear elastic modulus (kPa)	24.8 ± 11.1	21.4 ± 9.2^**^	19.2 ± 9.4	13.8 ± 7.5^**^	21.6 ± 15.1	21.7 ± 18.1	*p* = 0.011	0.261
Effect size	d=	0.338	d=	0.640	d=	0.003		

*Data are presented as mean ± SD.*

* *p < 0.05,*

** *p < 0.01, significant difference between PRE and POST*.

Moreover, a significant interaction effect was found for shear elastic modulus (*F* = 5.3, *p* = 0.11, and ${\text{η}}_{\text{p}}^{2}$ = 0.261), whereas the *post-hoc* test showed a significantly decreased shear elastic modulus after ROLL (*p* < 0.01 and *d* = 0.338) and Muscle Belly (*p* < 0.01 and *d* = 0.640); however, there was no significant change in MTJ (*p* = 0.977 and *d* = 0.003). According to the *post-hoc* test, no significant difference was found in absolute change in shear elastic modulus between ROLL and Muscle Belly (*p* = 0.25 and *d* = 0.461).

**Figure 1:**
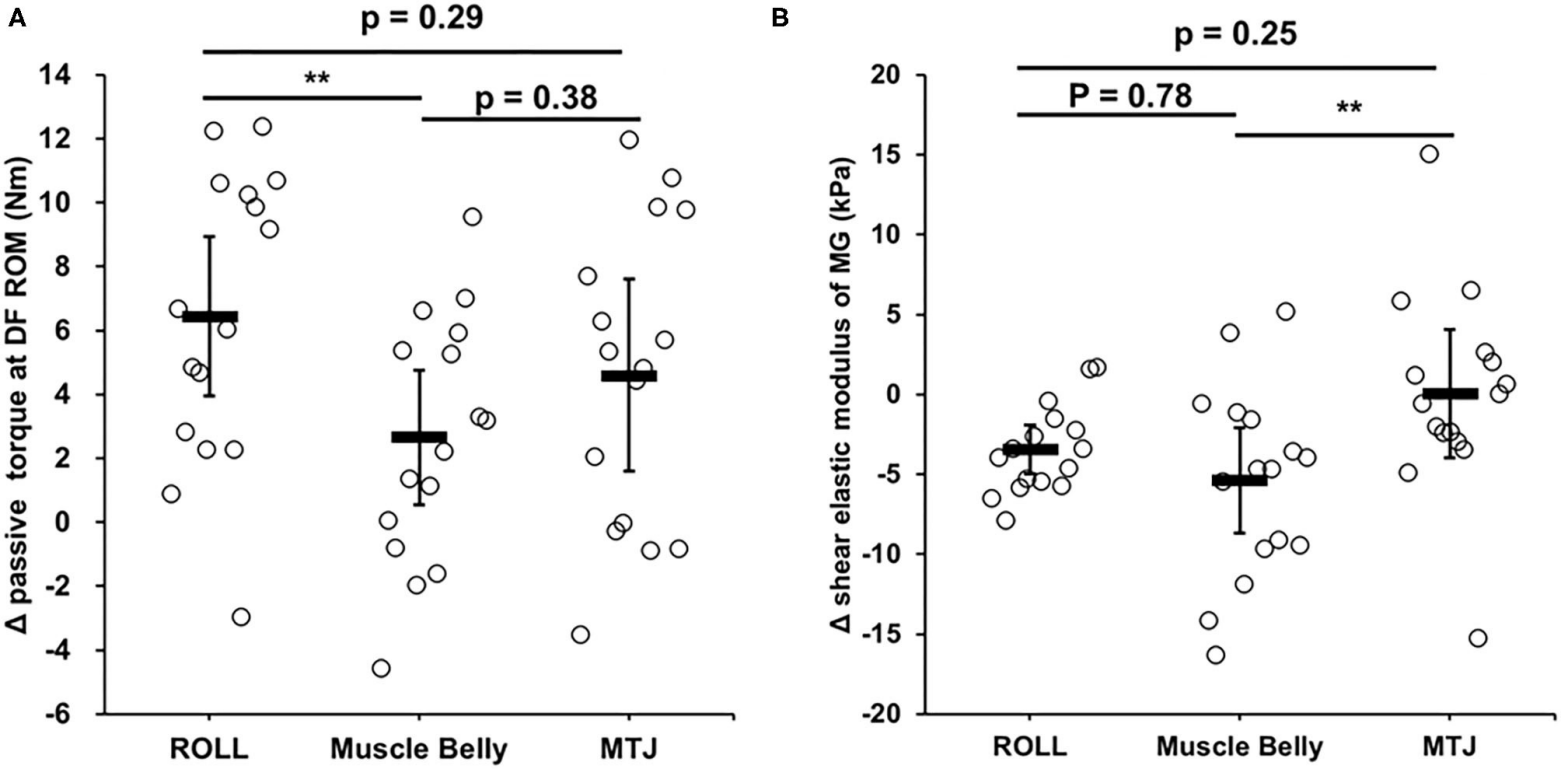
Normalized changes in passive torque at dorsiflexion range of motion (DF ROM) **(A)** and shear elastic modulus **(B)** from before (PRE) to immediately after intervention in three conditions, namely, vibration rolling, vibration on muscle belly, and vibration on the muscle-tendon junction (MTJ). ^**^p < 0.01.

Figure 1 shows the absolute change in passive torque at DF ROM and shear elastic modulus from before intervention to after intervention. The *post-hoc* test revealed absolute changes in passive torque at DF ROM in ROLL as significantly higher than Muscle Belly condition (*p* < 0.01, *d* = 0.93), whereas no significant differences were found between ROLL and MTJ or Muscle Belly and MTJ conditions.

Results for MVC-ISO, MVC-CON, and drop jump height are presented in detail in Table 2. Two-way repeated measures ANOVA showed no significant interaction effect (*F* = 0.88, *p* = 0.43, and $\eta_{\text{p}}^{2}$ = 0.05) and main effect of time (*F* = 0.05, *p* = 0.83, $\eta_{\text{p}}^{2}$ < 0.01) for MVC-ISO torque. Regarding the MVC-CON torque, a significant main effect was found for time (*F* = 4.99, *p* = 0.04, and $\eta_{\text{p}}^{2}$ = 0.227); however, there were no significant changes from PRE to POST in all conditions. As for drop jump height, a significant interaction effect was found (*F* = 4.25, *p* = 0.02, and $\eta_{\text{p}}^{2}$ = 0.200), whereas there were no significant changes from PRE to POST in all conditions.

**Table 2:** Changes in maximal voluntary isometric contraction torque (MVC-ISO), concentric torque at 30°/s (MVC-CON), and single-leg drop jump height before (PRE) and immediately after vibration rolling (ROLL) or vibration on the muscle belly (Muscle Belly) or vibration on muscle-tendon junction (MTJ) intervention (POST).

	**ROLL**		**Muscle Belly**		**MTJ**		**Interaction effect**	
	**PRE**	**POST**	**PRE**	**POST**	**PRE**	**POST**	***P-*value**	** $\eta_{\text{p}}^{2}$ **
MVC-ISO (Nm)	161.3 ± 26.8	160.4 ± 24.7	159.0 ± 22.1	157.6 ± 22.3	159.0 ± 26.5	160.7 ± 26.6	0.425	0.049
Effect size	d=	0.035	d=	0.065	d=	0.064		
MVC-CON (Nm)	129.8 ± 18.9	126.0 ± 16.0	129.5 ± 16.0	126.9 ± 14.1	128.4 ± 18.8	125.2 ± 18.5	0.922	0.005
Effect size	d=	0.218	d=	0.171	d=	0.172		
Drop Jump (cm)	19.9 ± 3.2	19.5 ± 2.7	19.9 ± 1.9	20.6 ± 2.3	20.2 ± 2.9	20.2 ± 2.7	0.022	0.2
Effect size	d=	0.149	d=	0.34	d=	0.002		

*Data are presented as mean ± SD*.

## Discussion

Our results showed that vibration with and without rolling, i.e., static compression of the muscle belly or MTJ could increase ROM, whereas vibration with rolling and static compression of the muscle belly could decrease muscle stiffness, but not after static compression of MTJ. In addition, vibration with and without rolling could not induce changes in muscle strength and jump performance. Results of this study showed that static compression for vibration FR intervention sufficiently increased ROM; however, vibration with rolling or static compression on the muscle belly was necessary to decrease muscle stiffness. To the best of our knowledge, this is the first study that thoroughly investigates the effects of vibration with and without rolling.

With regard to DF ROM changes, it was found to be significantly increased after the vibration FR intervention, which is consistent with that of previous studies (Cheatham et al., 2018; García-Gutiérrez et al., 2018; Lee et al., 2018). In addition, our results showed static compression of the muscle belly or MTJ with vibration, which could expand findings from a previous study showing that static compression of myofascial trigger points could increase the pain pressure threshold (Wilke et al., 2018). However, no significant differences were found in the increased DF ROM between vibration with and without rolling. Thus, if the goal is to increase ROM, static compression of vibration could sufficiently increase ROM since FR intervention with rolling is difficult to perform. However, previous studies showed the cross-transfer effect of FR and vibration FR (García-Gutiérrez et al., 2018; Nakamura et al., 2021a), and it was the possibility that the familiarization trial on the non-intervention side could increase the DF ROM on the intervention side. Further study is needed to investigate the cross-education effect of vibration with and without rolling, i.e., static compression of the muscle belly or MTJ on ROM.

Our results showed that passive torque was increased after vibration with and without rolling, inducing the modification in stretch tolerance. A previous study suggested that increased ROM after vibration FR intervention could occur due to the changes in pain perception (Cheatham et al., 2018), which supported our results. In addition, the change in passive torque at DF ROM in the ROLL condition was significantly higher than that in the muscle belly condition (Figure 1A). Thus, the change mechanism in pain perception is unclear; however, vibration with rolling or static compression of MTJ could possibly significantly change the pain perception and stretch tolerance.

Interestingly, the shear elastic modulus of MG was decreased after vibration with rolling and static compression of the muscle belly. Previous studies also reported that FR intervention for MG did not change the muscle hardness (Yoshimura et al., 2020) and shear elastic modulus (Nakamura et al., 2021b). Conversely, our findings showed that vibration with rolling and static compression of the muscle belly, which is assumed to induce greater changes in mechanoreceptors (Behm and Wilke, 2019), promoted a decrease in shear elastic modulus of MG. In addition, previous studies hypothesized that musculoskeletal structures respond to vibration because the tissue was required to adapt to or modulate the muscle tonicity to accommodate the wave of vibration (Musumeci, 2017; Germann et al., 2018). Similarly, with vibration application, Konrad et al. (2020) investigated the effect of percussive massage treatment at 53 Hz and suggested that percussive massage treatment could decrease muscle stiffness (Konrad et al., 2020). Therefore, vibration application, especially in the muscle belly area, not on the MTJ area, is assumed to decrease muscle stiffness, and a future study is needed to clarify the change mechanism of shear elastic modulus after local vibration application.

Surprisingly, no significant changes were observed in the muscle strength (MVC-ISO and MVC-CON) and drop jump height after vibration with rolling and static compression, which is partly consistent with those presented in previous studies (García-Gutiérrez et al., 2018; Lee et al., 2018). Theoretically, mechanical vibration applied to the muscle belly or tendon can elicit a tonic vibration reflex contraction of the target muscle (Germann et al., 2018). This mechanism is stimulated by a sequence of rapid muscle stretching that occurs when applying vibration and triggering muscle spindles, thereby causing an involuntary production of strength (Pamukoff et al., 2014; Germann et al., 2018). However, no significant changes were found in muscle strength and jump performance after vibration intervention. This study used vibration at 48 Hz; however, its effect on muscle strength and jump performance widely differs depending on the vibration frequency. Moreover, future studies should investigate the effect of different vibration frequencies on muscle strength and performance. Also, in drop jump measurement, joints other than the ankle joint might be involved. Therefore, further studies are needed to investigate the effect of vibration with rolling or static compression for other muscles on the drop jump height and other performance parameters.

Previous studies have pointed out that poor ROM (Witvrouw et al., 2001; Backman and Danielson, 2011) and increased muscle stiffness (Watsford et al., 2010; Pickering Rodriguez et al., 2017) are the risk factors for sports injuries. In addition, some previous studies showed that increased muscle stiffness associated with antagonist muscle contractions could inhibit joint movement, resulting in higher energetic/metabolic costs (Ueno et al., 2018; Blazevich, 2019). Thus, static stretching is commonly used to improve ROM and muscle stiffness, but the major problem with static stretching during the warm-up routine is decreased muscle strength and athletic performance caused by certain duration static stretching intervention (Kay and Blazevich, 2012; Behm et al., 2016). Our results showed that vibration with rolling or static compression of vibration on muscle belly could be effective to improve ROM and muscle stiffness without adverse effects of muscle strength and athletic performance. Especially in clinical application, previous studies showed that the muscle stiffness in patients with patellar tendinopathy (Zhang et al., 2017) or patients with Medial Tibial Stress Syndrome (Akiyama et al., 2016) was higher than healthy control participants. Although the participants were not athletes but physically inactive male individuals, vibration with rolling and static vibration on muscle belly could decrease muscle stiffness. Since static compression of vibration on muscle belly is safer and easier than a vibration in rolling, the static compression of vibration on muscle belly is recommended in rehabilitation settings.

Some limitations of this study are worth noting. Only the acute effects of vibration intervention on ROM and muscle stiffness had been investigated in this study. To the best of our knowledge, previous studies have investigated chronic effects of FR intervention without vibration (Smith et al., 2019; Kiyono et al., 2020); however, no study has yet determined the chronic effects of vibration with and without rolling on ROM and muscle stiffness; thus, this should be addressed in future studies. Furthermore, our participants were not athletes but physically inactive male individuals. Therefore, it is recommended to prescribe vibration with rolling or static vibration on muscle belly to decrease muscle stiffness and increase ROM in a clinical population needing rehabilitation. Moreover, we did not measure the effect of vibration on tendon property in addition to passive muscle property. Therefore, the effect of vibration intervention on muscle and tendon properties should also be investigated.

## Conclusion

Our results showed that local vibration application with rolling and static compression could increase ROM and modify stretch perception, i.e., stretch tolerance without changing muscle strength and jump performance. In addition, vibration with rolling and static compression of vibration on the muscle belly could decrease the shear elastic modulus of MG.

## Data Availability Statement

The raw data supporting the conclusions of this article will be made available by the authors, without undue reservation.

## Ethics Statement

The studies involving human participants were reviewed and approved by the Ethics Committee of the Niigata University of Health and Welfare, Niigata, Japan (Procedure #18304), and has complied with the Declaration of Helsinki requirements. The patients/participants provided heir written informed consent to participate in this study.

## Author Contributions

MN contributed to study design and data collection, drafted the manuscript, and made critical revision to the manuscript. SS, RK, RY, YM, KYas, and KYah contributed to data collection and made critical revisions to the manuscript. AK contributed to study design and data analysis, and made critical revision to the manuscript. All authors approved the final version of the manuscript and agreed to be accountable for all aspects of the work.

## Funding

This study was supported by JSPS KAKENHI with grant number 19K19890 (MN) and the Austrian Science Fund (FWF) project P 32078-B (AK).

## Conflict of Interest

The authors declare that the research was conducted in the absence of any commercial or financial relationships that could be construed as a potential conflict of interest.

## Publisher's Note

All claims expressed in this article are solely those of the authors and do not necessarily represent those of their affiliated organizations, or those of the publisher, the editors and the reviewers. Any product that may be evaluated in this article, or claim that may be made by its manufacturer, is not guaranteed or endorsed by the publisher.

## References

[B1] Akazawa N. Okawa N. Kishi M. Nakatani K. Nishikawa K. Tokumura D.. (2016). Effects of long-term self-massage at the musculotendinous junction on hamstring extensibility, stiffness, stretch tolerance, and structural indices: a randomized controlled trial. Phys. Ther. Sport 21, 38–45. 10.1016/j.ptsp.2016.01.003 27428533

[B2] Akiyama K. Akagi R. Hirayama K. Hirose N. Takahashi H. Fukubayshi T. (2016). Shear modulus of the lower leg muscles in patients with medial tibial stress syndrome. Ultrasound Med. Biol. 42, 1779–1783. 10.1016/j.ultrasmedbio.2016.03.010 27129903

[B3] Backman L. J. Danielson P. (2011). Low range of ankle dorsiflexion predisposes for patellar tendinopathy in junior elite basketball players: a 1-year prospective study. Am. J. Sports Med. 39, 2626–2633. 10.1177/0363546511420552 21917610

[B4] Baumgart C. Freiwald J. Kühnemann M. Hotfiel T. Hüttel M. Hoppe M. W. (2019). Foam rolling of the calf and anterior thigh: biomechanical loads and acute effects on vertical jump height and muscle stiffness. Sports 7:27. 10.3390/sports7010027 30669477 PMC6359537

[B5] Behm D. G. Blazevich A. J. Kay A. D. Mchugh M. (2016). Acute effects of muscle stretching on physical performance, range of motion, and injury incidence in healthy active individuals: a systematic review. Appl. Physiol. Nutr. Metab. 41, 1–11. 10.1139/apnm-2015-0235 26642915

[B6] Behm D. G. Wilke J. (2019). Do self-myofascial release devices release myofascia? Rolling mechanisms: a narrative review. Sports Med. 49, 1173–1181. 10.1007/s40279-019-01149-y 31256353

[B7] Blazevich A. J. (2019). Adaptations in the passive mechanical properties of skeletal muscle to altered patterns of use. J. Appl. Physiol. 126, 1483–1491. 10.1152/japplphysiol.00700.2018 30412028

[B8] Cheatham S. W. Stull K. R. Kolber M. J. (2018). Comparison of a vibration roller and a nonvibration roller intervention on knee range of motion and pressure pain threshold: a randomized controlled trial. J. Sport Rehabil. 28, 39–45. 10.1123/jsr.2017-0164 28787233

[B9] Cohen J. (1988). Statistical Power Analysis for the Behavioral Sciences. Hillsdale: Routledge.

[B10] Gajdosik R. L. Allred J. D. Gabbert H. L. Sonsteng B. A. (2007). A stretching program increases the dynamic passive length and passive resistive properties of the calf muscle-tendon unit of unconditioned younger women. Eur. J. Appl. Physiol. 99, 449–454. 10.1007/s00421-006-0366-7 17186300

[B11] García-Gutiérrez M. T. Guillén-Rogel P. Cochrane D. J. Marín P. J. (2018). Cross transfer acute effects of foam rolling with vibration on ankle dorsiflexion range of motion. J. Musculoskelet. Neuronal Interact. 18, 262–267. 29855449 PMC6016502

[B12] Germann D. El Bouse A. Shnier J. Abdelkader N. Kazemi M. (2018). Effects of local vibration therapy on various performance parameters: a narrative literature review. J. Can. Chiropr. Assoc. 62, 170–181. 30662072 PMC6319432

[B13] Hirata K. Kanehisa H. Miyamoto N. (2017). Acute effect of static stretching on passive stiffness of the human gastrocnemius fascicle measured by ultrasound shear wave elastography. Eur. J. Appl. Physiol. 117, 493–499. 10.1007/s00421-017-3550-z 28161870

[B14] Huang S. Y. Di Santo M. Wadden K. P. Cappa D. F. Alkanani T. Behm D. G. (2010). Short-duration massage at the hamstrings musculotendinous junction induces greater range of motion. J. Strength Cond. Res. 24, 1917–1924. 10.1519/JSC.0b013e3181e06e0c 20543728

[B15] Kay A. D. Blazevich A. J. (2012). Effect of acute static stretch on maximal muscle performance: a systematic review. Med. Sci. Sports Exerc. 44, 154–164. 10.1249/MSS.0b013e318225cb27 21659901

[B16] Kiyono R. Onuma R. Yasaka K. Sato S. Yahata K. Nakamura M. (2020). Effects of 5-week foam rolling intervention on range of motion and muscle stiffness. J. Strength Cond. Res. 10.1519/JSC.0000000000003757 33044364

[B17] Konrad A. Glashüttner C. Reiner M. M. Bernsteiner D. Tilp M. (2020). The acute effects of a percussive massage treatment with a hypervolt device on plantar flexor muscles' range of motion and performance. J. Sports Sci. Med. 19, 690–694. 33239942 PMC7675623

[B18] Konrad A. Močnik R. Titze S. Nakamura M. Tilp M. (2021). The influence of stretching the hip flexor muscles on performance parameters. A systematic review with meta-analysis. Int. J. Environ. Res. Public Health. 18:1936. 10.3390/ijerph18041936 33671271 PMC7922112

[B19] Konrad A. Tilp M. (2014). Increased range of motion after static stretching is not due to changes in muscle and tendon structures. Clin. Biomech. 29, 636–642. 10.1016/j.clinbiomech.2014.04.013 24856792

[B20] Lee C. L. Chu I. H. Lyu B. J. Chang W. D. Chang N. J. (2018). Comparison of vibration rolling, nonvibration rolling, and static stretching as a warm-up exercise on flexibility, joint proprioception, muscle strength, and balance in young adults. J. Sports Sci. 36, 2575–2582. 10.1080/02640414.2018.1469848 29697023

[B21] Mizuno T. Matsumoto M. Umemura Y. (2013). Viscoelasticity of the muscle-tendon unit is returned more rapidly than range of motion after stretching. Scand. J. Med. Sci. Sports 23, 23–30. 10.1111/j.1600-0838.2011.01329.x 21564309

[B22] Morales-Artacho A. J. Lacourpaille L. Guilhem G. (2017). Effects of warm-up on hamstring muscles stiffness: cycling vs foam rolling. Scand. J. Med. Sci. Sports 27, 1959–1969. 10.1111/sms.12832 28124382

[B23] Musumeci G. (2017). The use of vibration as physical exercise and therapy. J. Funct. Morphol. Kinesiol. 2:17. 10.3390/jfmk2020017

[B24] Nakamura M. Ikezoe T. Takeno Y. Ichihashi N. (2011). Acute and prolonged effect of static stretching on the passive stiffness of the human gastrocnemius muscle tendon unit in vivo. J. Orthop. Res. 29, 1759–1763. 10.1002/jor.21445 21520263

[B25] Nakamura M. Konrad A. Kiyono R. Sato S. Yahata K. Yoshida R.. (2021a). Local and non-local effects of foam rolling on passive soft tissue properties and spinal excitability. Front. Physiol. 12:702042. 10.3389/fphys.2021.702042 34248682 PMC8267519

[B26] Nakamura M. Onuma R. Kiyono R. Yasaka K. Sato S. Yahata K.. (2021b). The acute and prolonged effects of different durations of foam rolling on range of motion, muscle stiffness, and muscle strength. J. Sports Sci. Med. 20, 62–68. 10.52082/jssm.2021.62 33707988 PMC7919347

[B27] Nakamura M. Sato S. Hiraizumi K. Kiyono R. Fukaya T. Nishishita S. (2020). Effects of static stretching programs performed at different volume-equated weekly frequencies on passive properties of muscle-tendon unit. J. Biomech. 103:109670. 10.1016/j.jbiomech.2020.109670 32035662

[B28] Nakamura M. Sato S. Kiyono R. Yahata K. Yoshida R. Fukaya T.. (2021d). Association between the range of motion and passive property of the gastrocnemius muscle-tendon unit in older population. Healthcare 9:314. 10.3390/healthcare9030314 33809115 PMC8000756

[B29] Nakamura M. Sato S. Kiyono R. Yoshida R. Yasaka K. Konrad A. (2021c). Comparison between foam rolling with and without vibration on passive and active plantar flexor muscle properties. J Strength Cond Res. 10.1519/JSC.0000000000004123 PMC7613848 34474432

[B30] Pamukoff D. N. Ryan E. D. Blackburn J. T. (2014). The acute effects of local muscle vibration frequency on peak torque, rate of torque development, and EMG activity. J. Electromyogr. Kinesiol. 24, 888–894. 10.1016/j.jelekin.2014.07.014 25169762

[B31] Phillips J. Diggin D. King D. L. Sforzo G. A. (2021). Effect of varying self-myofascial release duration on subsequent athletic performance. J. Strength Cond. Res. 35, 746–753. 10.1519/JSC.0000000000002751 30024480

[B32] Pickering Rodriguez E. C. Watsford M. L. Bower R. G. Murphy A. J. (2017). The relationship between lower body stiffness and injury incidence in female netballers. Sports Biomech. 16, 361–373. 10.1080/14763141.2017.1319970 28553879

[B33] Reiner M. M. Glashüttner C. Bernsteiner D. Tilp M. Guilhem G. Morales-Artacho A.. (2021). A comparison of foam rolling and vibration foam rolling on the quadriceps muscle function and mechanical properties. Eur. J. Appl. Physiol. 121, 1461–1471 10.1007/s00421-021-04619-2 33638016 PMC8064982

[B34] Sato S. Hiraizumi K. Kiyono R. Fukaya T. Nishishita S. Nunes J. P.. (2020a). The effects of static stretching programs on muscle strength and muscle architecture of the medial gastrocnemius. PLoS ONE 15:e0235679. 10.1371/journal.pone.0235679 32645095 PMC7347101

[B35] Sato S. Kiyono R. Takahashi N. Yoshida T. Takeuchi K. Nakamura M. (2020b). The acute and prolonged effects of 20-s static stretching on muscle strength and shear elastic modulus. PLoS ONE 15:e0228583. 10.1371/journal.pone.0228583 32027694 PMC7004320

[B36] Smith J. C. Washell B. R. Aini M. F. Brown S. Hall M. C. (2019). Effects of static stretching and foam rolling on ankle dorsiflexion range of motion. Med. Sci. Sports Exerc. 51, 1752–1758. 10.1249/MSS.0000000000001964 30817716

[B37] Sullivan K. M. Silvey D. B. Button D. C. Behm D. G. (2013). Roller-massager application to the hamstrings increases sit-and-reach range of motion within five to ten seconds without performance impairments. Int. J. Sports Phys. Ther. 8, 228–236. 23772339 PMC3679629

[B38] Ueno H. Suga T. Takao K. Tanaka T. Misaki J. Miyake Y.. (2018). Potential relationship between passive plantar flexor stiffness and running performance. Int. J. Sports Med. 39, 204–209. 10.1055/s-0043-121271 29287284

[B39] Watsford M. L. Murphy A. J. Mclachlan K. A. Bryant A. L. Cameron M. L. Crossley K. M.. (2010). A prospective study of the relationship between lower body stiffness and hamstring injury in professional Australian rules footballers. Am. J. Sports Med. 38, 2058–2064. 10.1177/0363546510370197 20595555

[B40] Weppler C. H. Magnusson S. P. (2010). Increasing muscle extensibility: a matter of increasing length or modifying sensation? Phys. Ther. 90, 438–449. 10.2522/ptj.20090012 20075147

[B41] Wiewelhove T. Döweling A. Schneider C. Hottenrott L. Meyer T. Kellmann M.. (2019). A meta-analysis of the effects of foam rolling on performance and recovery. Front. Physiol. 10:376. 10.3389/fphys.2019.00376 31024339 PMC6465761

[B42] Wilke J. Müller A. L. Giesche F. Power G. Ahmedi H. Behm D. G. (2020). Acute effects of foam rolling on range of motion in healthy adults: a systematic review with multilevel meta-analysis. Sports Med. 50, 387–402. 10.1007/s40279-019-01205-7 31628662

[B43] Wilke J. Vogt L. Banzer W. (2018). Immediate effects of self-myofascial release on latent trigger point sensitivity: a randomized, placebo-controlled trial. Biol. Sport 35, 349–354. 10.5114/biolsport.2018.78055 30765920 PMC6358529

[B44] Witvrouw E. Bellemans J. Lysens R. Danneels L. Cambier D. (2001). Intrinsic risk factors for the development of patellar tendinitis in an athletic population. A two-year prospective study. Am. J. Sports Med. 29, 190–195. 10.1177/03635465010290021201 11292044

[B45] Yoshimura A. Schleip R. Hirose N. (2020). Effects of self-massage using a foam roller on ankle range of motion and gastrocnemius fascicle length and muscle hardness: a pilot study. J. Sport Rehabil. 29, 1171–1178. 10.1123/jsr.2019-0281 32050162

[B46] Zhang Z. J. Ng G. Y. F. Lee W. C. Fu S. N. (2017). Increase in passive muscle tension of the quadriceps muscle heads in jumping athletes with patellar tendinopathy. Scand. J. Med. Sci. Sports 27, 1099–1104. 10.1111/sms.12749 27539811

